# PReS-FINAL-2061: Abatacept in therapy of juvenile idiopathic arthritis in real clinical practice

**DOI:** 10.1186/1546-0096-11-S2-P73

**Published:** 2013-12-05

**Authors:** SV Arsenyeva, IP Nikishina, MI Kaleda, DL Alekseev, LG Medyntceva, SR Rodionovskaya

**Affiliations:** 1 Paediatric, Federal State Budgetary Institution "Research Institute of Rheumatology" under Russian Academy of Medical Science, Russian Federation; 2 Paediatric, Central Children Hospital FMBA, Moscow, Russian Federation

## Introduction

Biologics are often used in therapy of DMARDs resistant JIA. The most complicated problem is choosing specific biological agent in particular case. AntiTNF inhibitors are well studied, but the application of Abatacept in real clinical practice hasn't been investigated enough.

## Objectives

Retrospective evaluation of the ABA therapy results in process of treating children with JIA.

## Methods

The analysis includes data of 60 patients with JIA who were getting ABA in 2010 - 2013. The average age of patients is 12,5 years (from 6 to 18 years). 21 (35%) patients suffered from active uveitis (2 patients had one eye affection, 19 had affection of both eyes). 10 (16%) patients were diagnosed as sJIA without current systemic features. Patients received ABA intravenously at a dosage of 10 mg/kg with 2 weeks interval initially (3 infusion) and following every month administration. Efficacy was evaluated in accordance with ACRpedi criteria. Ocular manifestations were evaluated by ophthalmologists.

## Results

43 (72%) patients continue ABA therapy, in 17 cases ABA was cancelled (in 3 cases due to infusion/allergy reactions, in 9 cases due to the inefficacy of application, in 5 cases due to some other reasons). 30% patient (18 cases) failed to comply with the infusion schedule due to organization problems.

The efficiency of ABA according to the ACRpedi criteria is demonstrated in Table 1.

**Table 1:** 

\months ACRpedi\	3 (n = 55)	6 (n = 54)	9 (n = 51)	12 (n = 43)	18 (n = 33)	24 (n = 24)	30 (n = 9)
30%	51%	13%	2%	2%	0	4%	0

50%	49%	63%	24%	12%	15%	8%	0

70%	0	24%	55%	44%	24%	17%	11%

90%	0	0	6%	35%	55%	63%	67%

The efficacy of ABA was increased significantly after 12 months of therapy. The regularity of infusions influences the higher effect, thus 70-90% improvement after 12 months were achieved predominantly in patients who had no deviations in infusion regimen (30 vs 4). ABA used as first-line biological agent more often shows 70-90% improvement after 12 months (27 vs 7 antiTNF-failers). Uveitis remission was identified in 53% of cases after 6 months, in 73% of cases - after 12 months, in 69% of cases - after 18 months, in 67% of cases - after 24 four months. 7 patients had adverse events (3 patients has post-infusion reactions, 2 patients (the both with early pauciarthicular onset) had uveitis de-novo, 1 patient had body weight gain, 1 patient had verruca vulgaris) but no serious events were observed.

## Conclusion

Long-term application of ABA is supposed to be highly efficient for treatment of patients with JIA, including patients with uveitis. The increase of positive effect ABA has continued to increase after 24 month period of therapy. The efficiency of ABA depends on the correct infusion regimen and it is higher in biologics-naïve patients. ABA showed a good safety profile in the medium-term period.

## Disclosure of interest

None declared.

